# Sex differences in coronary artery plaque composition detected by coronary computed tomography: quantitative and qualitative analysis

**DOI:** 10.1007/s12471-019-1234-5

**Published:** 2019-02-08

**Authors:** F. Plank, C. Beyer, G. Friedrich, M. Wildauer, G. Feuchtner

**Affiliations:** 1 Department of Cardiology and Angiology, Innsbruck Medical University, Innsbruck, Austria; 2 Department of Radiology, Innsbruck Medical University, Innsbruck, Austria

**Keywords:** Coronary artery disease, Sex differences, Computed tomography angiography, Plaque morphology, Atherosclerosis

## Abstract

**Background:**

Sex differences in the calculation of coronary heart disease risk have been analysed extensively. However, data on coronary plaque morphology diverge. We analysed plaque characteristics in patients with suspected coronary artery disease (CAD) and defined prognostic factors using coronary computed tomography angiography (CCTA).

**Methods:**

A total of 6,050 consecutive patients underwent CCTA and were enrolled in the registry. Patients with known CAD were excluded. The patients were propensity score matched (1:1 male:female) for age and known coronary risk factors. Coronary arteries were evaluated for stenosis, plaque types (non-calcified, mixed and calcified) and high-risk plaque features (napkin-ring sign, low-attenuation plaque, spotty calcifications, positive remodelling). Clinical follow-up was performed.

**Results:**

A total of 1,050 patients (525 female, 525 male) in matched cohorts were selected for analysis. CCTA showed significantly higher calcium scores for males (mean 180.5 vs 67.8 AU, *p* < 0.0001) and a higher rate of CAD (66.0% vs 34.1%, *p* < 0.0001). In a total of 16,800 segments, males had significantly more plaques (861 vs 752, *p* < 0.0001) with a significantly larger proportion of calcified plaques, while females had more mixed and non-calcified plaques (33.5% vs 24.4%, *p* = 0.006 and 24.1% vs 13.6%, *p* = 0.22, respectively). After a mean follow-up of 5.6 years, major adverse cardiac event (MACE) rate was 5.3% in male and 1.9% in female patients (*p* < 0.05). The relative odds ratio for high-risk plaque features to predict MACE was higher in females.

**Conclusion:**

Based on a higher relative risk for women with high-risk plaque features, the findings of our study support the increased importance of a differentiated qualitative plaque analysis to improve the risk stratification for both sexes.

## What’s new?

- Male patients had a markedly higher rate of adverse findings on coronary computed tomography angiography (CCTA).
- CT showed a greater calcified plaque burden in males but a larger proportion of mixed and non-calcified plaques in females.
- There was a higher prevalence of high-risk plaque features in males, but a higher relative odds ratio for major adverse cardiac events in females.
- Sex differences in qualitative plaque analysis using CCTA may improve risk stratification.

## Introduction

Men have a significantly higher risk of developing coronary artery disease (CAD) than women [1]. Nevertheless, it is a misperception that females are ‘protected’ against cardiovascular disease (CVD); the risk merely appears to rise 7–10 years later due to exposure to endogenous oestrogens, as supported by study results evaluating CVD risk in women with early menopause [2] or oestrogen deficiency [3].

Still, CVD is the major cause of death in women. Sex differences between males and females are often overlooked and underappreciated in scientific studies involving the cardiovascular system. The same is true regarding the prevalence and presentation of acute coronary syndromes (ACS), which in females occur more often in the absence of obstructive CAD [4] due to smaller vessel size, less collateral flow, lower coronary flow reserve, more vascular stiffness and differences in remodelling [5, 6].

Non-invasive assessment of the vasculopathic differences in qualitative coronary stenosis and plaque parameters provide relevant information to improve risk stratification. Taking into consideration that the non-calcified plaque is more often associated with ACS than is the calcified plaque, identifying different plaque type morphologies and high-risk plaques prone to rupture may have an impact on predicting ACS and risk stratification [7].

Coronary computed tomography angiography (CCTA) has proved to be sensitive in the detection of early atherosclerosis [8] and the prediction of adverse cardiac events [9, 10], but large cohorts for differentiated qualitative plaque analysis are scarce and results diverging [11–14]. Recent reports underline the potential of CCTA to identify high-risk plaques based on quantitative analysis of plaque morphology.

## Materials and methods

### Study design and population

**Fig. 1:**
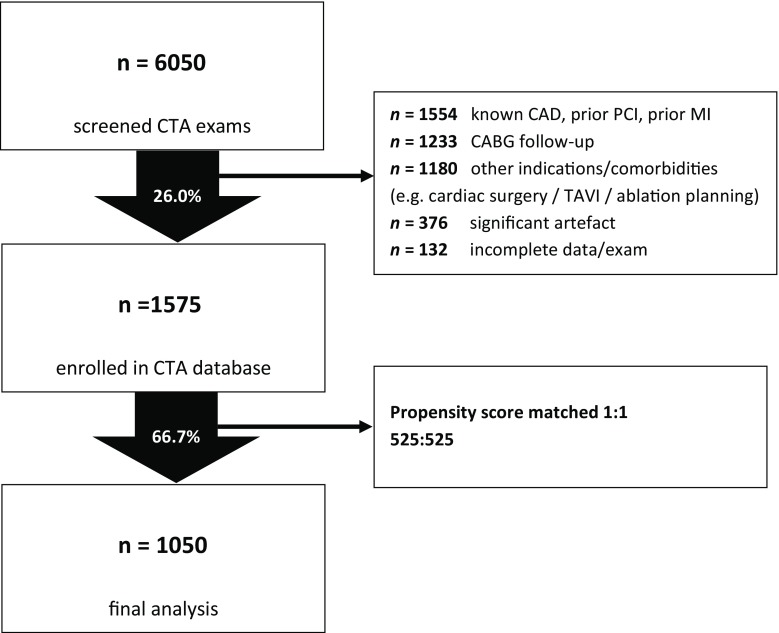
Flow chart describing patient selection (*CAD* coronary artery disease, *CABG* coronary artery bypass graft, *CTA* computed tomography angiography, *MI* myocardial infarction, *PCI* percutaneous coronary intervention, *TAVI* transcatheter aortic valve implantation)

All patients with suspected CAD referred for CCTA between 2006 and 2016 were prospectively included in a local registry. The study was approved by our local institutional review board; the requirement for patient informed consent forms was waived. Exclusion criteria for this study were: known CAD or prior revascularisation (including percutaneous coronary intervention, PCI, or coronary artery bypass graft, CABG), prior myocardial infarction, severe renal dysfunction (glomerular filtration rate <45 ml/min/1.73 m^2^), chest radiation or chemotherapy, non-diagnostic image quality (involving proximal or mid-segments). A flow chart of patient recruitment is provided in Fig. 1.

### Outcome and follow-up data collection

Follow-up was performed for all patients by reviewing medical records from the local system and referring hospitals were contacted for adverse events. Our centre is a tertiary-care, university hospital and is the only clinic providing invasive coronary angiography (ICA) and cardiac surgery in the province (covering specialised care for approx. 800,000 inhabitants). Additionally, the health care information system is connected to the central secondary care hospitals, expanding access to patient information. Data on ICA procedures and results, consecutive coronary revascularisation procedures (either PCI or CABG) were collected. The primary endpoint was a major adverse cardiac event (MACE) defined as a composite of ACS (including unstable angina, non-ST-elevation myocardial infarct (NSTEMI) and STEMI) and cardiac death by in-hospital documentation and/or post-mortem histology report. The secondary endpoint was revascularisation (PCI or CABG).

### CCTA examination

Two different CT scanners were used: a 64-slice scanner (Somatom Sensation 64, Siemens) with a detector collimation of 64 × 0.75 mm and a rotation time of 0.33 s until 2009; thereafter CCTA examinations (40.6%) were performed using a first-generation, 128-slice, dual-source CT scanner (Somatom Definition FLASH, Siemens).

An initial non-contrast ECG-gated calcium score (CCS) CT scan was performed and the Agatston score was calculated followed by CCTA. In patients examined with a 64-slice CT scanner, retrospective ECG gating was applied. Patients examined with the 128-DSCT equipment were scanned in either prospective ECG-triggered mode if they had a regular heart rate <65 bpm (70% of RR interval) and >65 bpm (40% of RR interval) or with retrospective ECG gating in patients with an irregular heart rate.

A contrast agent with an iodine concentration of 370 mg/ml was injected (flow rate 4–6 ml/s followed by 40 cc saline bolus); scans were triggered into arterial phase using a bolus tracking technique at a threshold of 100 Hounsfield units (HU) in the ascending aorta. Contrast volume varied between 65 and 120 ml in accordance with the individual patient’s body mass index and scan characteristics. All images were reconstructed with 0.75 mm slice width.

### CT image analysis

Coronary CT images were evaluated on a dedicated 3D post-processing software by two independent observers (2 years and 8 years of experience) and a consensus reading obtained in case of unclear findings. Only data sets without relevant artefacts leading to any non-diagnostic segment were included.

A standardised CCS was calculated for all patients. Contrast-enhanced CCTA evaluation included coronary stenosis divided into no stenosis, non-obstructive <50%, intermediate 50–70% and obstructive >70%. All plaques were documented and classified according to their morphology (1 calcified; 2 predominantly calcified; 3 predominantly non-calcified; 4 non-calcified).

Total plaque load was quantified using the segment involvement score (SIS, total number of segments with any plaque) according to the 16-segment American Heart Association classification and the non-calcified SIS (ncSIS), including non-calcified plaques only (type 4).

### High-risk plaque features

All lesions were evaluated regarding predefined high-risk features of vulnerable plaques [18].

- *Low-attenuation plaque*: The largest possible circle was drawn in the region of lowest attenuation, while omitting areas affected by artefacts or adjacent to calcifications. Plaques below 60 HU average were considered to be of low attenuation.
- The *remodelling index* was calculated as the ratio of the maximal cross-sectional vessel diameter at the plaque and its closest proximal (or distal, e. g. in case of an ostial lesion) normal reference diameter. A ratio of greater than 1.1 was considered relevant.
- *Spotty calcification* was defined as calcification (<3 mm) within a non-calcified plaque.
- *Napkin–ring sign*: If a plaque had a high-density outer rim (>200 HU) and an inner hypodense area (<150 HU) on a minimum of three stacked axial slices (1 mm). Adjacent calcifications/artefacts were excluded.

Follow-up was performed using patient chart check-ups. Information on clinical outcome was documented, including ACS (unstable angina, NSTEMI and STEMI), all-cause and cardiac mortality as well as revascularisation (PCI and CABG).

### Invasive coronary angiography

All diagnostic and therapeutic coronary angiographic procedures were performed at our centre. Obstructive CAD was defined as stenosis >70% or fractional flow reserve (FFR) <0.8 in cases of unclear visual grading. In cases of borderline stenosis without FFR validation, additional non-invasive ischaemia testing is required according to the local standard operating procedure in non-high-risk stenosis on CCTA before elective revascularisation.

### Statistical analysis

All statistical analyses were performed using SSPS software (V21.0, SPSS Inc.). Quantitative variables are expressed as means ± standard deviation, categorical variables as absolute values and percentages. A *p-*value of less than 0.05 was considered significant. Mean differences between parametric data were tested with Student’s *t*-test for two samples or one-way ANOVA for more, chi-square test or Fisher’s exact test for categorical data. Odds ratios were calculated using binary logistic regression.

### Propensity score matching

Matching factors were determined a priori and all variables forced into propensity scoring using logistic regression. Factors entered included age, male sex, hypertension, dyslipidaemia, diabetes mellitus, family history of premature cardiac death, smoking and body mass index. A nearest-neighbour approach using 1:1 matching was performed on site.

## Results

### Study cohort

Of 6,050 screened patients, 1,050 propensity score matched patients were selected for inclusion in the analysis. The mode of recruitment is depicted in Fig. 1; the demographics are outlined in Tab. 1.

**Table 1:** Study demographics

	Before matching 1,575 patients			After matching 1,050 patients		
	Male	Female		Male	Female	
*n*	853 (54.2%)	722 (45.8%)	*p*-value	525	525	*p*-value
Age (years)	56.6 ± 12.1	58.8 ± 11.4		58.3 ± 11.2	58.7 ± 11.0	0.57
BMI (kg/m^2^)	27.0 ± 4.1	25.8 ± 4.8	*<0.0001*	26.5 ± 3.4	26.1 ± 4.6	0.11
Smoking	290 (34.0%)	160 (22.2%)	*<0.0001*	213 (40.6%)	187 (35.6%)	0.12
Hypertension	365 (42.3%)	318 (44.0%)	0.13	271 (51.6%)	289 (55.1%)	0.15
Family history	214 (25.1%)	245 (33.9%)	*<0.0001*	193 (36.8%)	215 (41.0%)	0.42
Dyslipidaemia	379 (44.4%)	303 (42.0%)	0.28	282 (53.7%)	277 (52.8%)	0.38
– TC (mmol/l)	5.1 ± 1.2	5.3 ± 1.2	0.88	5.1 ± 1.1	5.4 ± 1.0	* 0.001*
– LDL (mmol/l)	4.2 ± 3.7	3.3 ± 2.5	* 0.001*	3.23 ± 0.9	3.3 ± 0.9	0.26
– HDL (mmol/l)	1.3 ± 0.4	1.6 ± 0.5	* 0.001*	1.4 ± 0.4	1.7 ± 0.5	*<0.0001*
Diabetes	74 (8.7%)	52 (7.2%)	0.37	54 (10.3%)	42 (8.0%)	0.33
– HbA_1C_ (%)	6.0 ± 1.2	5.8 ± 1.0	0.17	6.0 ± 1.3	5.0 ± 1.0	0.09
Creatinine (µmol/l)	79.6 ± 44.0	79.4 ± 42.2	0.95	79.0 ± 17.6	78.9 ± 15.0	0.39
Aspirin						
– Yes	138 (16.2%)	88 (12.2%)	0.08	98 (18.7%)	63 (12.0%)	0.09
– No	399 (46.8%)	296 (41%)		305 (58.1%)	288 (54.9%)	
– Unknown	316 (37.0%)	338 (46.8%)		122 (23.2%)	174 (33.1%)	
Statin						
– Yes	93 (10.9%)	65 (13.2%)	0.14	63 (12.0%)	41 (7.8%)	0.23
– No	445 (52.2%)	320 (64.9%)		388 (73.9%)	410 (78.1%)	
– Unknown	315 (36.9%)	337 (46.7%)		74 (14.1%)	74 (14.1%)	

*BMI* body mass index, *HDL* high-density lipoprotein, *LDL* low-density lipoprotein, *TC* total cholesterol

### Coronary plaque and stenosis quantification

Male patients had significantly worse CCTA findings than females. After adjusting for coronary risk factors, there was a significant difference in CCS (median 11.6 (0–174.4) vs 0 (0–12.5), *p* < 0.0001), prevalence of any CAD (66.0 vs 34.1%, *p* < 0.0001) and high-grade stenosis (27.5 vs 11.9%, *p* < 0.0001).

Plaque analysis showed a significantly higher proportion of overall affected segments in males (861 vs 752, *p* < 0.0001) with a higher proportion of calcified plaques (62% vs 42.4%, *p* < 0.0001) compared to females. Interestingly, females had significantly more mixed and non-calcified plaques (33.5% vs 24.4%, *p* = 0.006 and 24.1% vs 13.6%, *p* = 0.22, respectively). The prevalence of high-risk plaque features was higher for male patients (mean 41.1% vs 23.8%, *p* < 0.0001).

**Fig. 2:**
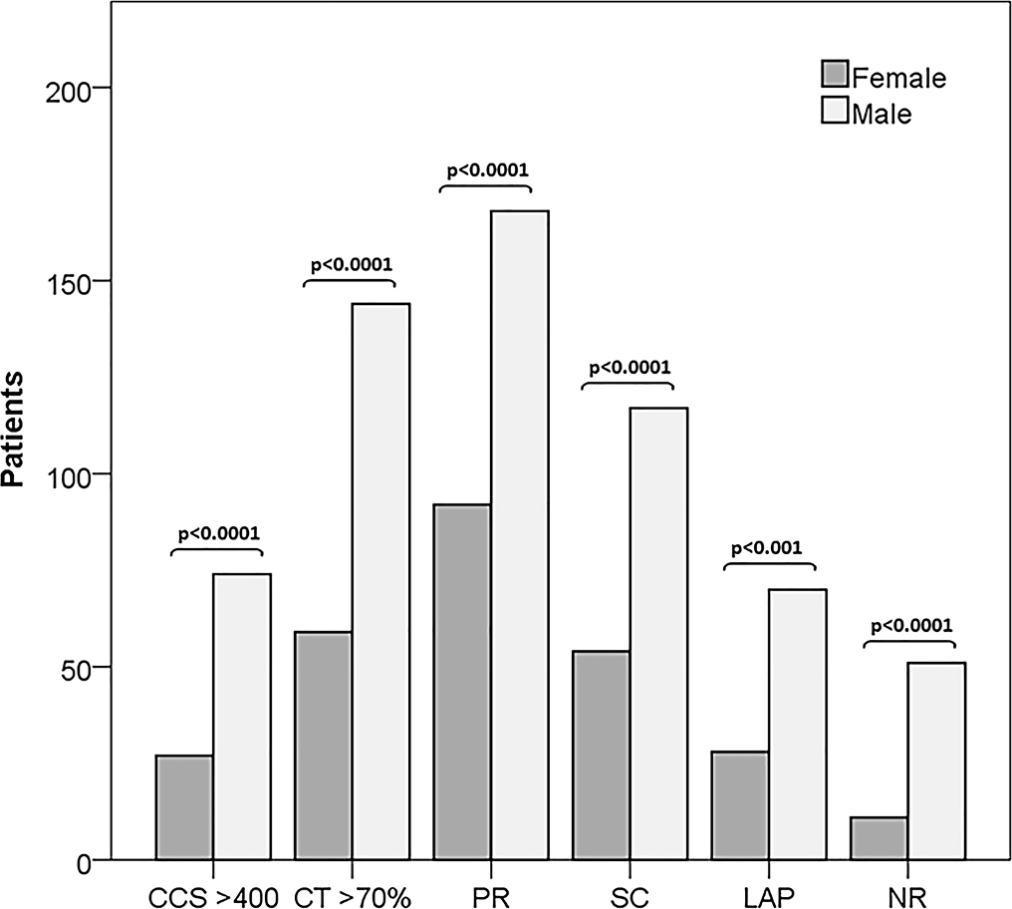
Bar chart showing the prevalence of CTA findings. Comparison of CT findings between females (*grey bars*) and males (*white bars*) (*CCS* coronary calcium score, *LAP* low-attenuation plaque, *NR* napkin-ring sign, *PR* positive remodelling, *SC* spotty calcification)

Sub-analysis of individual high-risk plaque features also showed a distinctly higher prevalence of every feature in male patients (Tab. 2, Fig. 2).

**Table 2:** CT findings

	Male	Female	
1,050 patients	525	525	*p*-value
CCS	11.6 (0–174.4)^a^	0 (0–12.5)^a^	*<0.0001*
CT stenosis			
– 0	231 (44.0%)	346 (65.9%)	*<0.0001*
– <50%	110 (21.5%)	88 (17.2%)	0.07
– 50–70%	43 (8.4%)	30 (5.9%)	0.11
– >70%	141 (27.5%)	61 (11.9%)	*<0.0001*
Plaques (16,800 segments)	861	752	*<0.0001*
– Calcified	534 (62.0%)	319 (42.4%)	*<0.0001*
– Mixed	210 (24.4%)	252 (33.5%)	* 0.006*
– Non-calcified	117 (13.6%)	181 (24.1%)	* 0.022*
Napkin-ring sign	51 (9.7%)	19 (3.6%)	*<0.0001*
LAP <60 HU	69 (13.1%)	29 (5.5%)	* 0.001*
– HU mean	40.4 ± 12.4	40.4 ± 9.4	0.99
Spotty calcification	120 (22.9%)	60 (11.4%)	*<0.0001*
Positive remodelling	172 (32.8%)	95 (18.1%)	*<0.0001*
Any HRP	216 (41.1%)	125 (23.8%)	*<0.0001*
ICA	163 (31.1%)	86 (16.4%)	*<0.0001*
ICA (31 days after CT)	74 (14.1%)	43 (8.2%)	* 0.004*
– <50%	44 (8.4%)	29 (5.5%)	0.053
– >50%	98 (18.7%)	33 (6.3%)	*<0.0001*
Revascularisation	75 (14.3%)	26 (5.0%)	*<0.0001*
– PCI	62 (11.8%)	19 (3.6%)	*<0.0001*
– CABG	18 (3.4%)	9 (1.7%)	0.10
MACE	28 (5.3%)	10 (1.9%)	* 0.005*
Death (all causes)	22 (4.2%)	17 (3.2%)	0.49
Death (cardiac)	25 (4.8%)	10 (1.9%)	* 0.015*
Follow-up (years)	5.8 ± 3.2	6.3 ± 3.1	0.058

*AU* Agatston units, *CABG* coronary artery bypass graft, *CCS* coronary calcium score, *HRP* high-risk plaque, *HU* Hounsfield units, *ICA* invasive coronary angiography, *LAP* low-attenuation plaque, *MACE* major adverse cardiac event, *PCI* percutaneous coronary intervention

^a^median (first and third quartile)

### Outcome

The total rate of all-cause mortality was 3.7% with significant differences in cardiac mortality of 4.8% for males versus 1.9% for females. MACE rate was higher in male than in female patients (5.3% vs 1.9%).

Patient follow-up was performed up to 11.5 years. Relevant information was available for at least 1 year in 975 patients (92.9%). After a mean period of 5.6 years, 31.1% of male patients were referred for invasive angiography (14.1% within 1 month after CCTA), while only 16.4% of female patients (5.0% within 1 month after CCTA) underwent ICA. After ICA, almost 50% of male patients underwent revascularisation therapy (80/163, 49.1%), but only approximately one third of female patients underwent PCI or CABG (28/86, 32.6%). FFR verification of haemodynamic relevance in obstructive stenosis was performed in 30 patients (12.1% of total ICA examinations), 51 patients additionally underwent nuclear stress imaging before ICA, and 46 patients (particularly earlier cases before 2008) underwent stress treadmill testing with significant ECG alterations.

**Fig. 3:**
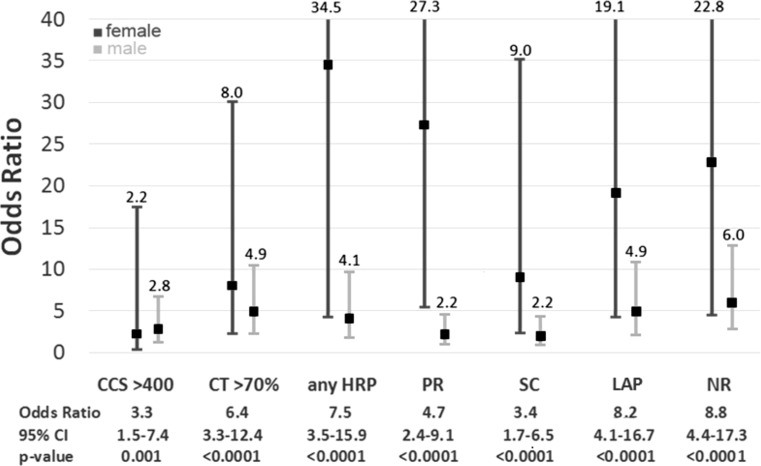
Odds radio (95% confidence interval, *CI*) for predicting acute coronary syndrome in female (*dark grey*) and male (*light grey*) patients by CT findings. Annotations below depict overall odds ratio (without separation by sex) (*CCS* coronary calcium score, *HRP* high-risk plaque, *LAP* low-attenuation plaque, *NR* napkin-ring sign, *PR* positive remodelling, *SC* spotty calcification)

The odds ratio for conventional CCTA findings and each high-risk plaque feature was significant in predicting revascularisation, MACE and cardiac death. While the event rate was not high enough to compare inter-sex differences, a trend towards a higher odds ratio for females could be observed. Positive remodelling was in fact significantly higher for female patients than for males (OR 27.3 (5.8–128.8) vs 2.2 (1.0–4.7)) (Fig. 3, Tab. 3).

**Table 3:** Odds ratio for coronary revascularisation and major adverse cardiac events divided into males and females

Odds ratio	Revascularisation		MACE	
	Male	Female	Male	Female
Napkin-ring sign	2.8 **** (1.6–4.8)	6.8 ** (1.6–28.9)	6.0 **** (2.8–12.9)	22.8 **** (4.7–111.6)
LAP <60 HU	2.6 **** (1.6–4.3)	5.8 ** (1.9–17.2)	4.9 **** (2.2–10.9)	19.1 **** (4.2–86.5)
Spotty calcification	3.1 **** (1.9–4.9)	5.2 **** (2.2–12.5)	2.0 (0.95–4.4)	9.0 *** (2.3–35.0)
Positive remodelling	3.3 **** (2.1–5.4)	5.8 **** (2.6–12.6)	2.2 * (1.0–4.7)	27.3 **** (5.8–127.7)
Any HRP	5.8 **** (3.3–10.2)	10.4 **** (4.2–25.6)	4.1 *** (1.8–9.7)	34.5 *** (4.3–280.3)
CT >70% stenosis	8.8 **** (5.2–15.0)	18.7 **** (7.7–45.2)	4.9 **** (2.3–10.5)	8.0 **** (2.1–29.9)
CCS >400 AU	3.1 **** (1.9–5.0)	12.5 **** (5.5–28.3)	2.8 * (1.2–6.8)	2.2 (0.3–17.6)

Pearson chi or Fisher exact for cells *n* < 5; odds ratio (binary logistic regression)

*AU* Agatston units, *CCS* coronary calcium score, *HRP* high-risk plaque, *LAP* low-attenuation plaque

* <0.05, ** <0.01, *** <0.001, **** <0.0001

**Fig. 4:**
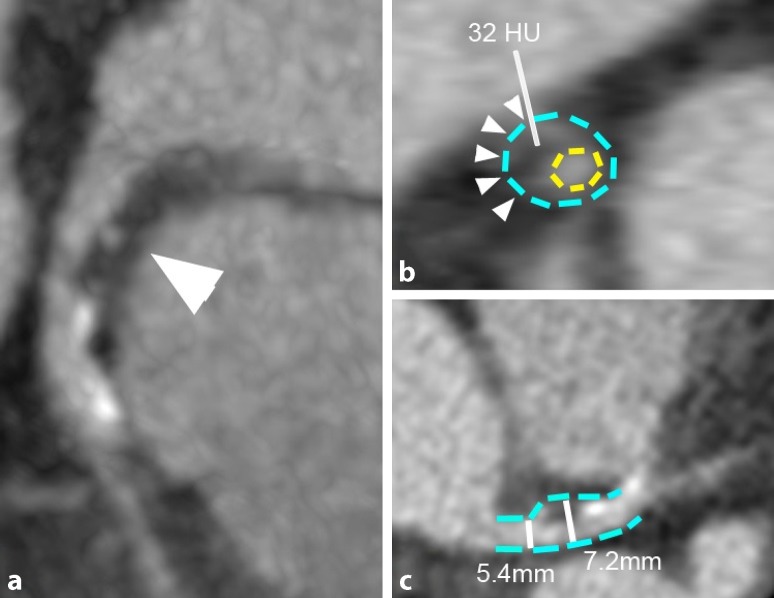
A 61-year-old patient with a history of smoking and diabetes. CTA revealed four high-risk plaque features in the distal left main/proximal left anterior descending artery (**a** *arrowhead*): low-attenuation plaque (**b** *annotation*) with napkin-ring sign (**b** *arrowheads*) and positive remodelling (**b**, **c** *dashed line*) and spotty calcification (**a**, **c**). The patient suffered from non-ST elevation myocardial infarction 2 months after CT and died of heart failure 3 years later

In a sub-analysis of the 42 MACE events, female patients had a lower CCS (63.3 AU vs 265.2 AU, *p* < 0.05) and all-grade stenosis (40% vs 57.1%, *p* < 0.05); however, females showed a trend towards a relatively higher prevalence of non-calcified plaques (33.3% vs 14.3%, *p* = 0.22) and presence of any high-risk plaque feature (80% vs 71.4%, *p* = 0.35) (Fig. 4).

## Discussion

Male sex is a major risk factor for CAD and is associated with increased plaque burden and stenosis severity on CCTA [1, 10]. Our results show a higher rate of calcified and total plaque burden as well as stenosis severity, which have been shown to independently predict cardiac events and worse outcome [9, 15, 16]. Our male population suffered from a significantly increased risk of MACE, while we observed a higher prevalence of high-risk plaque features, in line with the previous literature [17].

On the other hand, female patients had a significantly larger proportion of non-calcified and lipid-rich plaques, considered an even better predictor of myocardial ischaemia [18, 19].

Developments in recent decades have vastly improved the outcome of myocardial infarction and early recognition of CAD. Recent data from the National Health and Nutrition Examination Surveys have shown that over the past two decades the prevalence of myocardial infarction in middle-aged men (35–54 years) has decreased. However, that of women of the same age has increased [20]. The clinical presentation of CAD and the interpretation of functional non-invasive diagnostic testing is less reliable in women than in men, especially in the age group below 55 years where the prevalence of CAD is still relatively low [21, 22].

The recent randomised CRESCENT trial highlighted that CCTA is more efficient in women than in men in terms of time to reach the final diagnosis and downstream testing. This is largely attributed to an increase in prognostic information obtained for women on CCTA, while men fare similarly in anatomical and functional testing [23, 24]. Comparable results were found in the randomised ROMICAT-II trial, which recommended an early CCTA strategy in the evaluation of chest pain, reporting safe application with a lower cumulative radiation dose and shorter hospital stay [17].

The reports on plaque analysis and mortality prediction are heterogeneous. In the largest CCTA population from the CONFIRM registry including 5,632 patients, a larger number of plaques of all types were found in men than in women [25]. In contrast, a South Korean analysis reported a 6- to 7‑fold higher odds ratio for calcified and mixed plaque burden in asymptomatic male patients but an equal non-calcified burden [11]. In a smaller cohort, a higher rate of fibrous and lipid plaques was detected in the left coronary system of male patients [10], while another study found a higher relative proportion of non-calcified than calcified plaques in women [26].

Our results suggest a similar distribution regarding the higher prevalence of calcified and mixed plaques in males but a significantly higher proportion of non-calcified plaques in females. This increased plaque prevalence may be relevant for sex-specific risk stratification. Two ACS were documented in the present female cohort below 50 years with a profound cardiovascular risk profile, high-grade stenosis and high-risk plaque features but CCS of 0 AU and 1.4 AU, respectively.

Regarding the clinical implication of these findings, our sub-analysis of qualitative CCTA parameters (including high-grade stenosis and high-risk plaque features) showed a relative but statistically non-significant higher odds ratio for women but not in CCS. Considering the importance of non-calcified plaque with regard to the prediction of ACS, medium-term follow-up of 13,092 asymptomatic patients using CCS found markedly higher Agatston scores in males but no difference in cardiovascular events between the sexes [10]. This becomes even more evident when comparing CCS to CTA. Up to a third of asymptomatic patients with a CCS of zero may have non-calcified plaques [27].

This raises the question as to whether the well-established CCS may be of different significance for men and women. Considering the higher relative rate of non-calcified plaques in women, a low CCS may therefore have a different implication. To the best of our knowledge, no study has directly compared CCS in men and women with regard to MACE.

Similarly, male sex proved as major risk factor in the development of high-risk plaque and all of its sub-categories. The observed relative risk of MACE, however, was (non-significantly) higher in women. In fact, sub-analysing positive remodelling showed a significantly higher odds ratio for MACE in females than in males.

Another topic raised in a report from the European Heart Survey on stable angina pectoris indicates that women are less likely to be referred for non-invasive ischaemia testing or invasive angiography and consequently undergo an interventional procedure [28]. We did not observe a difference in referrals for ICA; however, subsequent revascularisation by PCI or CABG was significantly less likely in women than in men, after adjusting for rate of high-grade stenosis.

### Limitations

Our derivation cohort consisted of a relatively young population (mean 58 years) with a low prevalence of CAD. All patients were referred with a clinical suspicion of cardiovascular heart disease, the majority with a low-to-intermediate risk of CAD, which may have resulted in a selection bias, considering different clinical symptoms and indications in men and women.

Although long-term clinical follow-up was performed (for up to 11 years), a low overall mortality (3.5%) and MACE rate (3.6%) was observed. This resulted in a non-significant difference in MACE odds ratio in the sub-analysis between the sexes.

## Conclusion

Overall, our study supports a possible under-recognition of cardiovascular heart disease in women and suggests that more weight should be given to qualitative coronary plaque analysis by CCTA in order to improve the risk stratification of patients.
